# Determination of Serum Alpha-1-Acid Glycoprotein Concentration and the Influence of Physiological and Gynecological Factors in Women Attending in Tertiary-Care Center in Mexico: A Pilot Study

**DOI:** 10.1155/mi/9998286

**Published:** 2025-07-28

**Authors:** Liliana García-Ortiz, Mariana Téllez-Araiza, José Gutiérrez-Salinas, Liliana Hernández-Figueroa, Juana Arellano-García, Erasmo Cordero-Martínez

**Affiliations:** ^1^School of Medicine, Postgraduate Department, Universidad Nacional Autónoma de México (UNAM), Mexico City, Mexico; ^2^Division of Genomic Medicine and Clinical Genetics, National Medical Center “20 Noviembre”, ISSSTE, Mexico City, Mexico; ^3^Laboratory of Immunopharmacology, National Institute of Respiratory Diseases (INER), Mexico City, Mexico; ^4^Laboratory of Biochemistry and Experimental Medicine, Division of Biomedical Research, National Medical Center “20 Noviembre”, ISSSTE, Mexico City, Mexico; ^5^Clinical Laboratory, National Institute of Respiratory Diseases (INER), Mexico City, Mexico; ^6^Research Unit, National Institute of Respiratory Diseases (INER), Mexico City, Mexico

## Abstract

Alpha 1-acid glycoprotein (AGP1) is recognized as a protein with an important immunomodulatory activity, which under certain circumstances its serum concentration is altered as a result of acute metabolic stress (e.g., infections) or chronic (e.g., obesity). This quality of altering its concentration according to the immunological circumstances of the individuals, for example, in women, in which hormonal variables, nutritional states, and/or pregnancy, has led to think that one protein can be used as a biomarker in cancer, immunological diseases, and other pathologies. The aim of the present study was to determine the concentration of AGP1 protein in serum samples of apparently healthy women and to associate this concentration with several variables, such as age, body weight, and other physiological variables, that may affect the concentration of this protein. A total of 656 apparently healthy women were included according to their clinical history, physical examination, and laboratory tests. The determination of AGP1 concentration was performed by nephelometry in serum samples. The results show that the overall mean serum concentration of AGP1 was 76.82 ± 18.63 mg/dL (range 37.95–175 mg/dL). This concentration is observed to be altered in women who presented morbid obesity (83.79 mg/dL; *p*  < 0.001); as well as in women who have had children (gestation). Our work reports for the first time the serum levels of AGP1 in Mexican women, as well as several physiological circumstances in which its concentration is modified, which should be considered if AGP1 is required as a biomarker, both in physiological and pathological circumstances.

## 1. Introduction

Alpha 1-acid glycoprotein (AGP1) also known as orosomucoid (ORM) is a serum component of the immunocalin family, with a molecular weight of 41–43 kDa and 183 amino acids, highly glycosylated at 45% of its total weight [1, 2]. It is mainly synthesized in the liver by hepatocytes, although other tissues, such as lung, colon, prostate, as well as other cell lines, such as macrophages, epithelial cells of mammary tissue, endothelial cells, adipocytes, granulocytes, and neoplastic cells, can express it [3–6].

Serum concentration of AGP1 in healthy individuals ranges between 0.5 and1.4 mg/mL [7, 8]. However, infectious and traumatic conditions, tumors, surgical procedures, metabolic alterations, and any condition that implies a state of stress can induce its expression and increase it by 1–10 times, depending on the severity and/or stage of the disease [9].

AGP1 has been described as a negative immunomodulatory protein, however, the mechanism by which it performs this immunomodulatory function is not exactly known. It has been described that it can suppress neutrophil and complement activation by acting competitively with specific receptors and molecules [4, 9, 10]. It may also modulate cytokine secretion (TNF, IL-6, and IL-12) and inhibit lipopolysaccharide (LPS)-induced T-cell proliferation by decreasing IL-2 synthesis, leading to a decrease in cellular mitogens, and has been described as being able to inhibit the secretion of other pro-inflammatory factors [3, 6, 9–12].

It has also been observed that the immunomodulatory activity of AGP1 depends on the degree of branching of its glucan groups [11, 12] or may be altered in various pathophysiological states. Thus, during an acute inflammatory process, the concentration of N-glucan groups may decrease, while in chronic inflammatory processes the branching of N-glucan groups may be modified and contain a higher concentration of fucose [8].

A pathophysiological state that produces chronic low-grade inflammation is obesity, in which adipocytes produce adipokines, such as adiponectin, leptin, IL-6, TNF alpha, resistin, angiotensinogen, C-reactive protein, and AGP1 in a chronic manner, through signaling elevated levels of glucose, insulin, and free fatty acids; thus, adipose tissue is considered a regulator of physiological processes, such as immunity and inflammation, where AGP1 is involved in these biological events by protecting adipose tissue from excessive inflammation and metabolic dysfunction [13, 14].

The age and gender of individuals, as well as their ethnicity, also prove to be important factors in the modulation of AGP1. Increasing age has been reported to be associated with a decrease in plasma proteins, however, for AGP1, this concept remains controversial [15–17].

Another factor that may influence the immunomodulatory activity of AGP1 is pregnancy, where hormonal, metabolic, and nutritional changes induce a decrease in AGP1, such as what happens in premature births, term births, or with the intake of oral contraceptives; however, in surgical interventions, such as childbirth, cesarean section, or abortions, a decrease in serum AGP1 levels is observed [18–20].

Advances in the field of proteomics, biochemistry, and clinical studies in humans show that AGP1 could be considered a novel serum biomarker for several diseases associated with inflammation, immunity, and cancer [21]. However, as we can see, serum AGP1 levels fluctuate, depending on the physiological and/or pathological state; therefore, having reference intervals in study populations allows for the correct interpretation of data when performing biological measurements.

In the absence of reference values for a protein or molecule, the transferability of reference ranges from one population to another is feasible, however, there is a risk of changing the interpretation of the data due to differences in race, genetics, age of inclusion, as well as environmental factors and analytical determinations that may change the interpretation of the data [22, 23].

Currently, there is no analysis of AGP1 or its association with different physiological states in our population, nor are there any reference intervals that can be compared with other populations. Considering the need and the fact that it is a protein of great importance, which could be used as a biomarker in a variety of diseases, including, for example, breast cancer, the aim of this study is to determine and analyze serum levels of AGP1 in a sample of healthy Mexican women.

## 2. Material and Methods

### 2.1. Patient Selection

During the period from January to May 2022, female voluntary blood donors over 18 years of age were included in the study, who attended the blood bank of our institution, where a complete medical examination was carried out to explore their general health; in such a way that their complete state of health will be certified. A medical history was taken for each patient; physical examination and taking blood samples for biochemical analysis including blood biometry, blood chemistry, lipid profile, and viral panel. All women signed an informed consent form authorized by the institution's ethics and research committees.

### 2.2. Study Groups

We selected 656 unrelated women; categorically assuring that, at the time of the study, none of the selected women were pregnant and/or that, in the last year prior to the study, they did not have an abortion, cesarean section, or the birth of any child by natural means. With the above, we ensured that the women included in the study did not have any stressful gynecological events.

The women were grouped according to various gynecological events; which are defined as follows:

a. Women without gynecological event: We refer to a woman who is nulligravid; that is, who has never had a pregnancy, so she does not have a history of a live child, abortion, cesarean section, or any other gynecological event.
b. Women with gynecological event: This group of women are defined as those who have become pregnant and who had any of the following conditions related to their pregnancy: (1) Childbirth group; group of women where their pregnancy (no matter the number), came to term and the birth of the product was exclusively vaginal. (2) Cesarean section group; group of women where their pregnancy (no matter the number); it came to term and the birth of the product was exclusively by cesarean section. (3) Abortion group; group of women where their pregnancy (no matter the number); she did not reach term because she exclusively had spontaneous abortion. (4) Two or more events group; group of women who had in their pregnancies (no matter the number), a combination of two or more of any of the gynecological events described above (e.g., cesarean section plus abortion, normal delivery plus abortion, etc.).

### 2.3. AGP1 Determination

Blood samples were collected using 5.0 mL tubes containing SST gel to separate serum which was obtained by centrifugation (3500 rpm for 15 min). Serum samples were collected and stored at −20 °C for analysis within 72 h.

The analysis of AGP1 concentration in serum samples was performed using the kinetic nephelometry technique [24]. Nephelometry is the method by which a specific antibody binds to its antigen (AGP1) and, thus,makes the solution in which they are found more turbid. This turbidity is determined using a suitable spectrophotometer [25]. AGP1 was detected using a commercial kit (Beckman Colter, Immage Immunochemistry Systems; AGP1), and following the instructions provided by the manufacturer, detection was performed using a Beckman Colter equipment, model Immage 800. The sensitivity of the assay was 51–117 mg/dL.

### 2.4. Statistical Analysis

Statistical analysis was performed using a database in Excel (Microsoft Corporation) and GraphPhad Prism version 4.0 for Windows (GraphPad Software, San Diego, CA, USA). A descriptive analysis of the variables was performed, the results were presented as frequency and percentage for categorical variables and means and/or averages ± standard deviation for continuous variables. For comparison between groups, Student's *t*-test was used for continuous variables and *X*^2^ for categorical variables. A *p* value < 0.05 was considered statistically significant.

## 3. Results

The general characteristics of the women studied are shown in Table 1. It can be observed that the average age is 37 years with a range of 18–67 years. 40% of them are married, 30% are single, and the rest are in a free union and/or divorced, widowed, or separated. Most of the women belong to a middle socio-economic level and their education is high school (73.21% and 72%, respectively). The above is important because these women belong to the healthcare system provided by our government; that is, our institution (Instituto de Seguridad y Servicios Sociales de los Trabajadores del Estado; ISSSTE). It provides services to a stratum of the population with a fixed income and who do not belong to a disadvantaged social class. The classification of the economy into low, middle, and high refers to the fact that the income they receive depends on their academic preparation and administrative position.

All women belong to the middle class; however, as previously mentioned, their income is commensurate with their academic level and job position. According to the World Bank and INEGI, the women's economic levels are: low (with incomes up to $8000 per capita); middle (up to $13,000 per capita); and high (>$13,000 per capita). According to the World Bank, the women in our study belong to the economic level upper-middle to high-income range [26, 27]. Thus, women who were grouped according to these economic levels (Table 1), did not present statistically significant differences in their average AGP1 concentration.

Considering the average body mass index (BMI), according to the World Health Organization definition [28], the body composition of most of the women studied was in the type I obesity range and according to their gynecological history only 36.6% of the women used contraceptives.

Likewise, the serum concentration of AGP1 was determined in all the women, which obtained an overall average of 76.82 ± 18.63 mg/dL (37.95–175) (Table 2).

**Figure 1:**
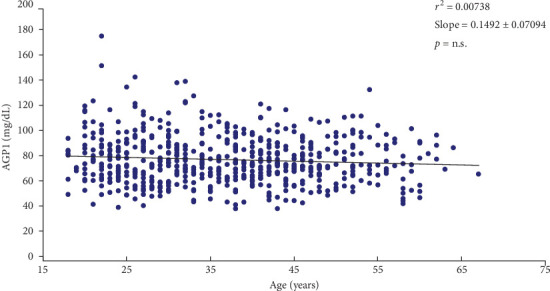
Age (years) vs. AGP1 (mg/dL) association. The figure shows a negative correlation between serum AGP1 concentration vs. age in all women.

Considering what has been reported by Lehallier et al.[29], serum AGP1 concentration may have changes in its concentration, dependent on the age of the subjects; an association curve between AGP1 concentration and age was carried out (Figure 1). The analysis values obtained show that there is no correlation between age and serum AGP1 concentration (slope: 0.1492 ± 0.07094; *r*^2^: 0.00738; *p*= not significant). However, when the women are grouped by age ranges (Table 2), it is observed that there is a higher mean concentration of AGP1 in women in the ranges <20 (81.5 ± 19.7); 51–55 (80.05 ± 18.81) > 61 (80.68 ± 10.89); showing no statistical differences between all ranges.

When an analysis is performed between serum AGP1 concentration and BMI of all women (Table 2), it is observed that there is a statistically significant difference between the serum AGP1 value of women with healthy weight compared with morbidly obese women (70.46 vs. 83.79 mg/dL, respectively; Student's *t*, *p*  < 0.001).

Due to the fact that some proteins present changes in their serum concentration depending on the pregnancy and hormonal status [30, 31], an analysis was performed according to BMI and serum AGP1 concentration in the group of women without and with gynecological event. As it can be seen in Table 3, there are statistically significant difference between serum AGP1 concentration average when comparing the group of women without gynecological event vs. the group of women with gynecological event.

Considering the women's use of contraceptives, they were grouped as follows: (A) women with gynecological events without contraceptive use; (B) women with gynecological events with contraceptive use; (C) women without gynecological events without contraceptive use; (D) women without gynecological events with contraceptive use.

Thus, in the groups of women with gynecological events (A vs. B), contraceptive use did not alter AGP1 concentrations compared to the group not using contraceptives: (75.61 ± 15.32 vs. 76.38 ± 17.52; *p*=0.82, respectively). Furthermore, in the groups of women without gynecological events (C vs. D), there was no statistically significant difference in AGP1 concentrations with or without contraceptive use. (77.23 ± 16.23 vs. 76.01 ± 16.46; *p*=0.92; respectively).

For the purposes of our study, all participating women were grouped by gynecologic events: those who did not present any gynecologic event; women who had exclusively childbirth, cesarean section, or abortion; and those who presented a mixture of two or more of the above gynecologic events (as described in material and methods). Thus, Table 4, section A, shows 203 women without gynecological events and 453 women with gynecological events; of which 129 presented exclusively childbirth; 123 exclusively cesarean section and 23 exclusively abortion. While 178 women had a mixture of two or more of some of the aforementioned events.

It is also observed that childbirth was the gynecological event that presented the highest ranges of the groups. When childbirth occurred exclusively, it had an average of 2.16 (range 1–5), while when it was associated with another event, the average was 1.29 with the same range.

On the other hand, the BMI of the participants was grouped considering the gynecological events (Table 4, section B). As can be observed, the average BMI of women presenting exclusively abortion is the lowest of all of them, and it is statistically significant when comparing this average, with the group of women having exclusively cesarean section and the group presenting two or more gynecological events (24.10 ± 5.02 [18.94–34.18] vs. 31.67 ± 10 [19.15–61.93] and 31.81 ± 8.78 [17.89–63.91]; *p*  < 0.05; respectively).

Similarly, the concentration of AGP1 in women who presented the gynecological events described above is also shown (Table 4, section C). In them, it can be observed that women belonging to the without gynecological event group, present the highest average AGP1 (81.02 ± 20.17; range 45.7–151.5 mg/dL); compared to the groups with the different gynecological events; with the exception of the group of women exclusively childbirth. On the other hand, it can be observed that women who presented exclusively abortions show the lowest average AGP1 concentration (58.22 ± 21.36; range 41.05–110.5 mg/dL); being this value statistically significant (*p*  < 0.05; Student's *t*-test); when compared to the other groups.

Considering that, serum concentration of AGP1 in women who presented pregnancy and that it ended in a childbirth or cesarean section, it can be verified that the concentration of this protein varies depending on the number of deliveries and cesarean sections, compared to women without any type of gynecological event. In this way we can corroborate that the average of AGP1 in women without gynecological events is 81.02 ± 20.16 mg/dL. This concentration is lower in women who presented 1–2 childbirth-cesarean sections (73.85 ± 19.16 mg/dL; *p*=0.007) as well as in those who presented 3–4 childbirth-cesarean sections (74.98 ± 18.44 mg/dL; *p*=0.043). However, in women who had ≥5 childbirth-cesarean, this difference in serum AGP1 concentration was not observed (83.55 ± 14.32 mg/dL; *p*=0.7). Nor was a statistically significant difference in serum AGP1 concentration observed between the groups of women with any number of childbirth-cesarean section; (1 to ≥5 childbirth-cesarean section).

**Figure 2:**
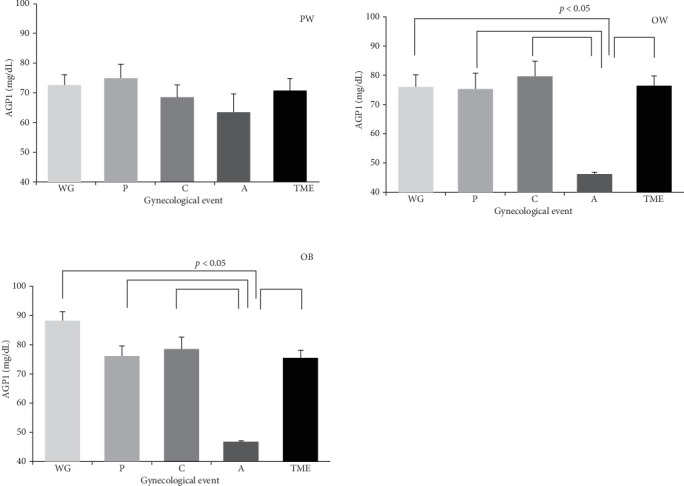
AGP1 concentration in diverse gynecological events. The gynecological events are associated according to BMI as shown: PW, proper weight women; OW, overweight; OB, obesity. Gynecological events are as follows: A, abortion; C, cesarean section; P, childbirth; TME, two or more events; WG, without gynecological event.

Finally, it was observed that women in the proper weight group, without gynecological event or with gynecological event associated exclusively with childbirth, cesarean section, or abortion and those who presented the mixture of two or more of these gynecological events (TME), did not have statistically significant differences, when comparing the averages of the serum concentration of AGP1 and its relationship with the gynecological event and BMI (Figure 2).

However, when comparing women in the overweight or obese group who presented exclusively with abortion, a statistically significant difference (*p*  < 0.05) was observed between them and the rest of the groups (Figure 2).

## 4. Discussion

Worldwide there are few reports in the literature related to the standard serum quantification of AGP1 in healthy population. AGP1 is considered an acute phase protein mainly involved in immunological biological processes and inflammatory responses, so its determination could serve as a serum biomarker in health and disease [32].

It is important for our study that serum AGP1 values are maintained at baseline; thus, the determination of the serum concentration of AGP1 that we have detected in Mexican women is the direct product of their baseline condition, at that specific time for this protein; since this group of women has at least 1 year since any of the gynecological events in which they were grouped (childbirth, abortion, cesarean section, a combination of them; or without any of these events) occurred. It is also noteworthy that, at the time of taking the blood samples, the women did not present any inflammatory, infectious, traumatic event, or cancer that was detected in the interview or by general laboratory blood tests.

This is the first report on serum levels of AGP1 in women of Mexican origin, where the average value is 76.82 + 18.63 mg/dL; similar to the average values reported for healthy women from the United States of America which is 73.9 + 16.8 mg/dL [33], and healthy adults from Denmark which is 78.0 mg/dL [34] or healthy adult monozygotic twins from Germany which is 90 mg/dL [35].

Recent research carried out by Lehallier et al. [29], indicates that, with senescence, the concentration of some proteins in the bloodstream does not present a linear ascending or descending pattern, as the case may be, but rather an undulating pattern.

This undulating behavior of serum AGP1 concentration was observed in our population (Table 2). Considering the average value of the entire group, it is observed that there are variations (although not statistically significant) in the serum concentration of AGP1, when women are grouped in periods of 5 years; with an apparently wave-like behavior in people in the age groups between <20 and 50 years, increasing again in people between 51 and 55 years of age and in those over 61 years of age.

Increased or decreased serum AGP1 concentration has been reported equally in several previous studies. Blain [33] and Störiko [35] reported an increase in serum values of this protein in women with increasing age. On the other hand, Veering et al. [15], reports that there are no serum variations in the concentration of AGP1 due to age. These controversial results on the concentration of AGP1 and its relationship with age should be investigated with greater attention. These bio-chronological findings will have to be considered if standards for the routine determination of this serum protein are to be made, when trying to distinguish between normal and abnormal serum values, which will also be influenced by the archetype of the population analyzed [34, 36].

Another important factor to highlight is the relationship between serum levels of AGP1 and the BMI of the women analyzed. Our results show that women present an increase in the general mean in serum AGP1 concentration, considering the increase in BMI; being statistically significant in the obese morbid group, where the maximum point is reached (Table 2). This behavior in serum AGP1 concentration is consistent with what was recently reported [37]. It should be noted that obesity is considered a systemic disease that causes chronic low-grade inflammation, caused by hypertrophy of adipocytes that release pro-inflammatory proteins [38, 39]. Adipose tissue has the ability to release pro-inflammatory components, such as cytokines; IL-6; TNF-alpha; and leptin. In addition, this tissue can express the respective receptors for these components, as well as have the ability to attract macrophages, neutrophils, and T-regulatory cells to the fat tissue itself and, thus promote in some way, the expression of the AGP1 protein, probably as an immunoregulatory factor. This is because it has also been reported that AGP1 can influence locally, avoiding the excessive inflammatory process caused by the conditions imposed by fat tissue [40]. It is clear that obesity is a complex disease that, in turn, produces other diseases that can affect the body's metabolism related to the general inflammatory and immunological process [41].

When analyzing our results, we observed that the serum concentration of AGP1 in women who presented a gynecological event was statistically lower when compared to the group that did not present any gynecological event (Table 3). The difference between the two groups does not seem to be affected by age, since we observed that there is no correlation between serum AGP1 concentration and age, as shown in Figure 1. This variation in serum AGP1 concentration due to a gynecological event has been previously described in Swedish women, showing a decrease in serum AGP1 concentration in pregnant women, compared to the control group that did not have pregnancy [30]. On the other hand, it has been reported that different gynecological events considered as “stressful” (e.g., cesarean section, preeclampsia, and recurrent miscarriage), have been shown to produce changes in some proteins related to inflammatory and immunological processes [37, 42, 43].

In our study, it was observed that in the different groups of gynecological events that were performed, there was a significant decrease in the serum concentration of AGP1 when compared to the without gynecological event group; This decrease was statistically relevant in the group that has abortion (Table 4). The causes of these variations in serum AGP1 concentration when a gynecological event occurs are not fully known and require further study. In this sense, it has been shown that a stressful acute gynecological event, such as recurrent miscarriage produces a decrease in acute phase proteins that participate in immunological, inflammatory, and blood clotting processes. In physiological pregnancy, these phenomena may be part of a regulatory response that is carried out during the invasion of the trophoblast to the endometrium; where it has been seen that when the trophoblast expands there is both an immunological and inflammatory response that can serve to sustain the mother's tolerance to the embryo [44, 45].

It is known that a person with a BMI above the healthy range presents a chronic low-grade inflammatory process permanently (as long as it is above the BMI) [46]. The excess of body fat tissue of these people causes the serum concentration of AGP1 to present alterations, which denote their participation in this chronic inflammatory process; probably regulating some of the pro-inflammatory factors, as well as some cell lines that participate in this process [46].

Considering the above, we think that a woman who has a chronic low-grade inflammatory process caused by her excess body fat; presents a greater inflammatory state when a gynecological event occurs. This condition alters the expression of AGP1; especially when abortion occurs and even more so, when the person shows excess body fat. It is striking that, despite the time that has elapsed since the last gynecological event; serum AGP1 levels remain altered, as demonstrated when compared to the serum levels of women without pregnancies.

In this regard, it has been described that during pregnancy some pro-inflammatory and immunological factors are modified; and they can persist for a long time after birth [43, 47]. In this regard, Ezeigwe et al., [48] showed that important serum proteins involved in inflammatory and immunological processes, such as C-reactive protein, D-dimer, and interleukin-6, show significant variations in women who have had one to five pregnancies, and these changes are visible and persistent until they are over 60 years of age. This is not the case in women who have not had gynecological events, where there are no significant variations in these proteins. This phenomenon can be thought of as an “immunological memory of pregnancy”; it may have an impact on a woman's overall health [49].

The need to develop reliable methods for detecting factors that regulate processes as complicated as immunological and inflammatory events is evident. The detection of key proteins that show these metabolic conditions must consider the bio-chronological characteristics of the population. Ethnicity, gender, age, as well as particular genetic characteristics, are aspects that must be considered in order for a given element to be qualified as a serum marker of the condition that needs to be evaluated.

We think that, since the AGP1 protein is part of the response system of both immunological and inflammatory processes, it may be useful as a complementary biomarker to other biochemical determinations (e.g., C-reactive protein; cytokines; complement system, etc.). The inclusion of routine determination of serum AGP1 concentration in the clinical practice is still controversial; however, having new serum markers that help in the evaluation of health status can be a very useful tool to show the clinical course of diseases that are associated with inflammatory and immunological processes. If this were possible, it would have a valuable diagnostic tool.

## 5. Conclusion

The present study shows that mean serum concentration of AGP1 in Mexican women is 76.82 ± 18.63 mg/dL (37.95–175) and that, under the influence of pathophysiological conditions, such as nutritional status or pregnancy, AGP1 shows statistically significant fluctuations.

This is the first report on the serum concentration of AGP1 in Mexican women, and could be considered as a reference in the search and/or standardization of serum biomarkers for diseases associated with inflammation and immunity.

## Figures and Tables

**Table 1:** Baseline characteristics of the women included in the study (*n* = 656).

Variables	Values
Age (y)^a^	36.95 ± 11.01 (18–67)
Married *n* (%)	—
Married	261 (40)
Single	196 (30)
Separate, widow, and divorced	95 (14.5)
Free union	103 (15.7)
Economy (%)	—
USD $ 8000.00 (low)	20%
USD $ 13,000.00 (middle)	73.21%
USD $ >13,000.00 (high)	6.80%
Education (%)	—
<Middle school	28%
>Middle school	72%
Weight (kg)^a^	69.99 ± 11.69 (51–120)
Height (cm)^a^	158.7 ± 10.73 (158–183)
BMI (kg/m^2^)^a^	31.54 ± 10.77 (16.26–85.71)
Systolic pressure (mmHg)	104 (96–117)
Diastolic pressure (mmHg)	69 (62–76)
Age at menarche (y)^a^	14 (12–16)
Age at menopause (y)^ab^	46.43 ± 2.31 (43–51)
Oral contraceptives users; *n* (%)	240 (36.6)

^a^mean ± SD (range).

^b^has corresponding.

**Table 2:** Serum AGP1 concentration (mg/dL), considering the various age and BMI ranges.

Global concentration of AGP1 = 76.82 ± 18.63 (37.95–175)						
	*n*	Mean	S.D.	Minimum	Maximum	*p* ^⁣^*∗*^^
Age (y)						
<20	31	81.5	19.7	49.25	119.5	—
21–25	89	79.29	22.26	39.05	175	—
26–30	102	76.61	20.76	40.4	142.5	—
31–35	83	79.06	20.61	43.4	139	—
36–40	91	75.6	16.7	37.95	112.5	—
41–45	95	74.92	17.6	38	121	—
46–50	71	74.63	15.3	42.6	109	—
51–55	47	80.05	18.81	51.5	132.5	—
56–60	34	70.2	17.69	41.9	101.5	—
>61	13	80.68	10.89	65.45	96.4	—
	—	—	—	—	—	n.s.
BMI (kg/m^2^)						
Underweight	18	74.84	16.87	58.55	104.5	—
Proper weight	181	70.46	17.88	41.05	115	—
Overweight	159	75.6	18.51	40.4	123	—
Obese I	115	77.46	18.84	37.95	119	—
Obese II	76	79.73	17.95	43.4	116.5	—
Morbidly obese	107	83.79*⁣*^*∗∗*^	20.48	56.2	151.5	—
	—	—	—	—	—	<0.05

*Note:* Variables are expressed as mean ± SD; range (minimum, maximum).

*⁣* ^*∗*^ANOVA analysis.

*⁣* ^*∗∗*^ *p* < 0.001 (Student-*t* test), proper weight vs. morbidly obese groups.

**Table 3:** BMI and AGP1 in women without and with gynecological event groups.

	Without gynecological event (*n* = 203)		With gynecological event (*n* = 453)	
	BMI (kg/m^2^)	AGP1 (mg/dL)	BMI (kg/m^2^)	AGP1 (mg/dL)
Mean	32.33	81.02	31.10	74.58*⁣*^*∗*^
S.D.	11.97	20.17	9.87	18.41
Minimum	16.26	45.70	17.89	37.95
Maximum	70.67	151.50	85.71	138

*Note:* Variables are expressed as mean ± SD; range (minimum, maximum).

*⁣* ^*∗*^ *p*  < 0.05 vs. without gynecological event group in each variable.

**Table 4:** Gynecological events: description of the all events evaluated (part A), and according to BMI (part B) or AGP1 (part C).

Variable	*n*	Mean	SD	Range	
A) Gynecological event					
Without event	203	—	—	—	—
Childbirth	129	2.16	1	1–5	—
Cesarean section	123	1.85	0.79	1–3	—
Abortion	23	1.27	0.64	1–3	—
Two or more events	178	—	—	—	—
Gestation	—	3.28	1.08	1–7	—
Childbirth	—	1.29	1.24	0–5	—
Cesarean section	—	1.13	0.92	0–4	—
Abortion	—	0.85	0.75	0–3	—

**Variable/** **gynecological event**	***n***	**Mean**	**SD**	**Minimum**	**Maximum**

B) BMI (kg/m^2^)					
Without event	203	32.33	11.97	16.26	70.67
Childbirth	129	30.80	11.41	18.01	85.71
Cesarean section	123	31.67^**a**^	10	19.15	61.93
Abortion	23	24.10	5.02	18.94	34.18
Two or more events	178	31.81^**a**^	8.78	17.89	63.91
C) AGP1 (mg/dL)					
Without event	203	81.02^**a**^	20.17	45.70	151.50
Childbirth	129	75.50^**a**^	18.64	44.60	112
Cesarean section	123	76.40^**a**^	19.67	37.95	138
Abortion	23	58.22	21.36	41.05	110.50
Two or more events	178	74.74^**a**^	16.16	40.40	110

^**a**^ *p*  < 0.05 vs. abortion group.

## Data Availability

The data that support the findings of this study are available from the corresponding author upon reasonable request.
